# *Gordonia bronchialis*–Associated Endophthalmitis, Oregon, USA

**DOI:** 10.3201/eid2505.180340

**Published:** 2019-05

**Authors:** Rene Choi, Luke Strnad, Christina J. Flaxel, Andreas K. Lauer, Eric B. Suhler

**Affiliations:** Casey Eye Institute, Portland, Oregon, USA (R. Choi, C.J. Flaxel, A.K. Lauer, E.B. Suhler);; Oregon Health and Science University, Portland (L. Strnad);; Veterans Administration Portland Health Care System, Portland (E.B. Suhler)

**Keywords:** Gordonia bronchialis, bacteria, actinomycetes, endophthalmitis, eye, eye-related infections, uveitis, Oregon, United States

## Abstract

*Gordonia bronchialis* is an aerobic actinomycetes that rarely causes infections in humans. Few reports describe *Gordonia* spp. causing eye-related infections. We report a case of chronic infectious endophthalmitis in Oregon, USA, associated with infection by *G. bronchialis.*

*Gordonia bronchialis* is an aerobic bacteria that rarely causes infections in humans. We report a case of chronic infectious endophthalmitis caused by infection with *G. bronchialis.*

A 63-year-old woman was referred to a uveitis service in Portland, Oregon, USA, because of a 10-month history of decreased vision in the left eye caused by 3 recurrences of anterior and intermediate uveitis that was refractory to topical corticosteroids. Her ocular history included cataract surgery with intraocular lens implants placed bilaterally 2.5 years before symptom onset. Her medical history included chronic obstructive pulmonary disease, diabetes mellitus type 2, and hypertension. A complete review of systems, including fevers, chills, night sweats, weight loss, or history of contacts with ill persons, was unremarkable.

Her visual acuity was 20/20, and she had hand motion vision at 2 feet in the right and left eyes. No afferent pupillary defect was appreciated, and intraocular pressures were normal bilaterally. In the left eye, anterior segment examination showed 4+ anterior chamber cell with a hypopyon and a diffuse white granular sheet along the posterior face of the intraocular lens implant. Visualization of the left fundus was precluded by 4+ vitreous cell and haze. B-scan ultrasonography showed extensive vitreous opacities and an attached retina (Figure).

**Figure:**
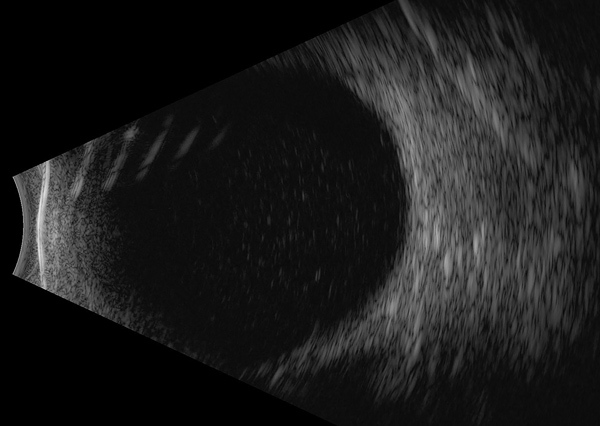
B-scan ultrasonography of the left eye of a 63-year-old woman with *Gordonia bronchialis*–associated endophthalmitis, Oregon, USA, showing dense opacities in the vitreous space.

Laboratory test results were negative for rapid plasma reagin, fluorescent treponemal antibody absorption, tuberculosis (Quantiferon-Gold TB; Quest Diagnostics, https://www.questdiagnostics.com), angiotensin-converting enzyme, rheumatoid factor, antinuclear antibody, and human leukocyte antigen B27. We obtained standard results for a complete blood count with differential counts, erythrocyte sedimentation rate, complete metabolic panel, and chest radiography.

The patient was given a diagnosis of presumptive chronic infectious endophthalmitis and underwent a pars plana vitrectomy and intraocular lens implant removal of the left eye. A vitreous sample was sent for pan-culture, cytologic analysis, flow cytometry, and broad-range PCR analyses with 16S rRNA gene sequencing. Results of cytology analysis and flow cytometry were negative for neoplastic processes. Culture analysis showed gram-positive bacilli but did not identify the species.

The 16S rRNA gene sequencing yielded positive results for *G. bronchialis*. We confirmed the bacterium to be *G. bronchialis* by using matrix-assisted laser desorption/ionization time-of-flight mass spectrometry. Antimicrobial susceptibility testing showed favorable MICs for amikacin, ceftriaxone, amoxicillin/clavulanic acid, and ciprofloxacin. Subsequently, the patient was treated with an intravitreal injection of 400 µg of amikacin to the left eye.

Two weeks after this intervention, visual acuity had improved to 20/100 in the left eye and ocular inflammation had resolved. However, 3 weeks later, the patient returned because of worsening symptoms, hand motion vision, and severe symptomatic recurrent anterior chamber and vitreous inflammation in the left eye. Intravitreal ceftazidime (2.25 mg) was administered to the left eye, and a 21-day course of oral moxifloxacin (400 mg/d) was prescribed. One week after completing her moxifloxacin regimen, her symptoms had improved, her best corrected visual acuity had improved to 20/40 in the left eye, and intraocular inflammation had resolved.

*Gordonia* spp. are gram-positive, weakly acid-fast aerobic actinomycetes that are ubiquitous in the environment; 29 species have been identified (*1*). Human infections are rare, although a few case reports of sternal wound infections, bloodstream and intravascular catheter related infections, and skin abscesses have been published (*2*). Optimal antimicrobial drug treatment for infection with *Gordonia* spp. is unknown. However, the organisms are generally believed to be susceptible to cephalosporins, aminoglycosides, and fluoroquinolones (*3*).

One previous case of *Gordonia* spp. playing a pathogenic role in an eye-related infection in a case of traumatic endophthalmitis secondary to infection with *G. sputi* has been reported (*4*). In contrast, the peculiarity of our case was the lack of obvious mode of transmission for *G. bronchialis* into the intraocular space. Seeding of the intraocular lens implant at the time of cataract surgery is possible, but unlikely, given the time lapse of 2.5 years between surgery and onset of symptoms. The patient also had no risk factors or history suggesting seeding from the bloodstream.

The course of our patient was of particular interest because the eye initially improved after vitrectomy and administration of intravitreal amikacin. However, a robust recurrence of inflammation occurred 3 weeks later, probably caused by incomplete treatment during initial therapy.

Although previously accurate identification of *Gordonia* spp. by using traditional culturing techniques has been challenging, advent of molecular biology techniques, such as 16S rRNA sequencing and matrix-assisted laser desorption/ionization time-of-flight mass spectrometry, has improved identification of aerobic actinomycetes (*3**–**8*). Because the organism was partially identified by culture and fully identified by multiple molecular techniques and responded clinically to targeted antimicrobial drugs, we believe this case was a pathogenic infection and not a nonpathogenic contaminant/colonizer.

We report a case of chronic infectious endophthalmitis caused by infection with *G. bronchialis.* Molecular methods have enabled successful identification of this organism, which is generally considered to have low virulence and be highly susceptible to antimicrobial drugs. A combination approach of pars plana vitrectomy and intraocular lens explantation with intravitreal and oral antimicrobial drugs might lead to a successful outcome.
